# The Curcumin Analogs 2-Pyridyl Cyclohexanone Induce Apoptosis via Inhibition of the JAK2–STAT3 Pathway in Human Esophageal Squamous Cell Carcinoma Cells

**DOI:** 10.3389/fphar.2018.00820

**Published:** 2018-08-21

**Authors:** Ying Wang, Pengjun Zhou, Shurong Qin, Dandan Xu, Yukun Liu, Wuyu Fu, Bibo Ruan, Li Zhang, Yi Zhang, Xiao Wang, Yuwei Pan, Sheng Wang, Haizhao Yan, Jinhong Qin, Xiaoyan Wang, Qiuying Liu, Zhiyun Du, Zhong Liu, Yifei Wang

**Affiliations:** ^1^Guangdong Provincial Key Laboratory of Bioengineering Medicine, Institute of Biomedicine, College of Life Science and Technology, Jinan University, Guangzhou, China; ^2^College of Food Science and Technology, Zhongkai University of Agriculture and Engineering, Guangzhou, China; ^3^Guangdong Food and Drug Vocational College, Guangzhou, China; ^4^School of Basic Courses, Guangdong Pharmaceutical University, Guangzhou, China; ^5^Cancer Center, Department of Surgery, Yale University, New Haven, CT, United States; ^6^Department of Pharmacy, Shenzhen People’s Hospital, The Second Clinical Medical College of Jinan University, Shenzhen, China; ^7^College of Medicine, Jinan University, Guangzhou, China; ^8^Interdisciplinary Graduate School of Medicine and Engineering, University of Yamanashi, Yamanashi, Japan; ^9^Institute of Natural Medicine and Green Chemistry, School of Chemical Engineering and Light Industry, Guangdong University of Technology, Guangzhou, China

**Keywords:** 2-pyridyl cyclohexanone, STAT3, Bcl-2, human esophageal squamous cell carcinoma, apoptosis

## Abstract

Multiple modifications to the structure of curcumin have been investigated with an aim to improve its potency and biochemical properties. Previously, we have synthesized a series of curcumin analogs. In the present study, the anticancer effect of 2-pyridyl cyclohexanone, one of the curcumin analogs, on esophageal carcinoma Eca109 and EC9706 cell lines and its molecular mechanisms were investigated. 2-Pyridyl cyclohexanone inhibited the proliferation of Eca109 and EC9706 cells by inducing apoptosis as indicated by morphological changes, membrane phospholipid phosphatidylserine ectropion, caspase 3 activation, and cleavage of poly(ADP-ribose) polymerase. Mechanistic studies indicated that 2-pyridyl cyclohexanone disrupted mitochondrial membrane potential, disturbed the balance of the Bcl-2 family proteins, and triggered apoptosis via the mitochondria-mediated intrinsic pathway. In 2-pyridine cyclohexanone-treated cells, the phosphorylation levels of JAK2 and STAT3 were dose-dependently decreased and p38 and p-ERK signals were notably activated in a dose-dependent manner. Moreover, we found that the addition of S3I-201, a STAT3 inhibitor, led to a decreased expression level of Bcl-2 in Eca109 cells. The chromatin immunoprecipitation assay demonstrated that STAT3 bound to the promoter of Bcl-2 in the Eca109 cells. Furthermore, the mutation of four STAT3 binding sites (−1733/−1723, −1627/−1617, −807/−797, and −134/−124) on the promote of Bcl-2 gene alone attenuated the transcriptional activation of STAT3. In addition, down-regulation of STAT3 resulted in less of transcriptional activity of STAT3 on Bcl-2 expression. These data provide a potential molecular mechanism of the apoptotic induction function of 2-pyridyl cyclohexanone, and emphasize its important roles as a therapeutic agent for esophageal squamous carcinoma.

## Introduction

Esophageal cancer is the eighth most common cancer worldwide. Esophageal cancer includes esophageal squamous cell carcinoma (ESCC) and esophageal adenocarcinoma (EAC). Its incidence is significantly affected by regional and ethnic differences (Enzinger and Mayer, 2003; Pennathur et al., 2013). The incidence and fatality rate of esophageal carcinoma in China is high (Liu et al., 2010). The 5-year survival rate of ESCC is only 10% (Ferlay et al., 2015). It is reported that 5% of all cancer deaths in 2012 were due to ESCC (McGuire, 2016). Furthermore, ESCC accounts for 80% of all cases of esophageal cancer worldwide, and is the predominant histological subtype (Colquhoun et al., 2015). ESCC is commonly diagnosed at an advanced stage because of the absence of early symptoms. Thus, clarification of its pathogenesis and new methods for its prevention, diagnosis, and treatment are urgently needed.

Signal transducers and activators of transcription (STATs) were originally discovered as DNA-binding proteins. They mediate interferon-dependent gene expression (Fu et al., 1992; Schindler et al., 1992a,b, Schindler, 2011). STAT3 protein is a key regulator of human cancers, contributing to proliferation, uncontrolled differentiation, survival, invasion, tumorigenesis, and resistance to chemotherapy (Buettner et al., 2002; Yu and Jove, 2004; Haura et al., 2005; Grivennikov et al., 2009; Wang et al., 2011; Yu et al., 2014; Huynh et al., 2017). It is reported that STAT3 is highly active in myeloma cell lines (Levy and Darnell, 2002), as well as in head and neck squamous cell (Sriuranpong et al., 2003; Adachi et al., 2012), breast (Behera et al., 2010; Lei et al., 2016), brain (Garg et al., 2017), gastric (Zhang et al., 2017), lung (Gritsko et al., 2006; Levy and Granot, 2006; Yu et al., 2009; Looyenga et al., 2012; Assi et al., 2014), and esophageal carcinomas (Liu et al., 2015). Moreover, the growing evidence has shown that activation of STAT3 plays a prominent role in cell growth and survival. Studies have shown that numerous genes which encode for Bcl-2, Bcl-xL, cyclins D1/D2, BIRC5, CDKN1A, MCL-1, and c-MYC proteins are downstream targets of STAT3 (Turkson and Jove, 2000; Bhattacharya et al., 2005; Milner et al., 2008; Yu et al., 2014; Abroun et al., 2015). Although abundant evidence suggests that STAT3 is an ideal target for cancer therapy, to date, effective therapeutic interventions to inhibit STAT3 and generate a potent antitumor effect clinically remain to be explored and developed (Yu et al., 2014).

Curcumin (diferuloylmethane) is a naturally occurring compound identified from *Curcuma longa*. It has many medicinal properties, such as antioxidant, antiproliferative, antiangiogenic, antitumorigenic, and anti-inflammatory properties (Chainani-Wu, 2003; Epstein et al., 2010). Curcumin has been explored as a prospective therapeutic agent for treatment of several cancers, such as head and neck squamous cell carcinoma (Chakravarti et al., 2010), colorectal carcinoma (Villegas et al., 2008; Patel and Majumdar, 2009), and pancreatic cancer (Glienke et al., 2010). It has been indicated that curcumin targets different biochemical pathways, such as those mediated by HER2 (Aggarwal et al., 2003), Wnt/β-catenin (Leow et al., 2010), Janus kinase (JAK)/STAT (Kim et al., 2003), and nuclear factor (NF)-κB (Epstein et al., 2010), to enhance its effect on cancer cells. Curcumin also targets cellular transformation, invasion, angiogenesis, and metastasis (Lin et al., 2010a; Seo et al., 2010). Therefore, the National Cancer Institute has listed curcumin as a third-generation cancer chemopreventive agent. Under physiologic condition, curcumin is not so stable and its absorbtion is not good through ingestion (Sharma et al., 2007). Many modifications of curcumin have been explored with an aim to improve its potency and biochemical properties (Fuchs et al., 2009; Ravindran et al., 2010).

We have synthesized a series of curcumin analogs in previous studies (**Table 1**). Prior to this experiment, we had preliminarily screened a series of synthesized drugs, from which 2-pyridyl cyclohexanone (No. 26) was chosen for further studies. In the present study, we performed an *in vitro* study to investigate the direct antitumor effect of one of the analogs, 2-pyridyl cyclohexanone, and its molecular mechanisms in esophageal carcinoma cell lines (Eca109 and EC9706). 2-Pyridyl cyclohexanone is a small molecular compound that has an obvious inhibitory effect on ESCC cells. The effects of 2-pyridine cyclohexanone on cell proliferation and apoptosis, with a particular focus on its possible influence on STAT3 status, were investigated.

**Table 1:**
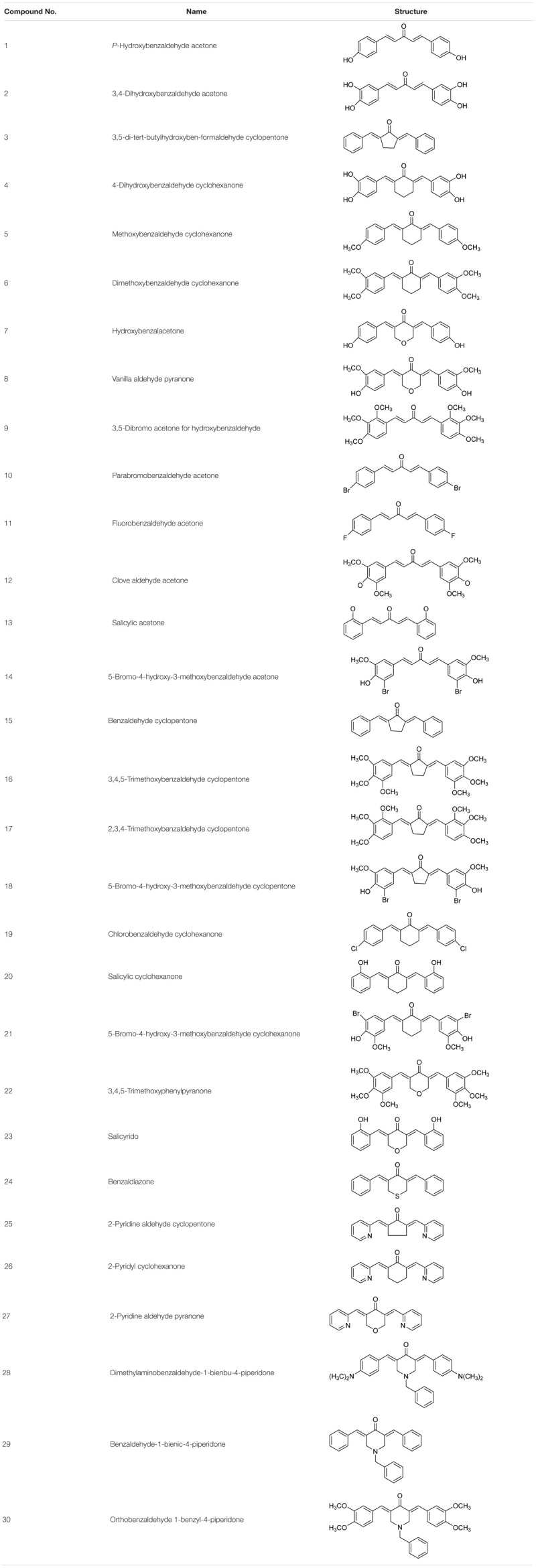
Chemical structures of the curcumin analogs.

## Materials and Methods

### Cell Culture

Eca109 and EC9706 cells were kindly provided by Cell Bank of the Chinese Academy of Sciences (Shanghai, China). The cells were cultured in Roswell Park Memorial Institute-1640 medium (Life Technologies, Rockville, MD, United States) or Dulbecco’s modified Eagle’s medium supplemented with 10% (v/v) heat-inactivated fetal bovine serum (Sigma-Aldrich, St. Louis, MO, United States) and 1% penicillin/streptomycin (Life Technologies, Rockville, MD, United States) at 37°C in a humidified atmosphere of 5% CO_2_.

### Reagents

2-Pyridyl cyclohexanone (>98% purity) was synthesized by Guangdong University of Technology (Guangzhou, China). S3I-201 (≥97% purity, high-performance liquid chromatography grade) was purchased from Sigma (Houston, TX, United States).

Antibodies against caspase-3 (#9662), poly(ADP-ribose) polymerase (PARP) (#9542s), Bcl-2 (#2870s), Bcl-xL (#2764), Bax (#2772s), Bid (#8762), p38 (#8690), p-p38 (#9211s), ERK (#4695), p-ERK (#T202), STAT3 (#9139), p-STAT3 (Tyr705) (#9145), JAK2 (#3230p), p-JAK2 (Tyr1007/1008) (#3776s), and glyceraldehyde-3-phosphate dehydrogenase (GAPDH) (#5174) were purchased from Cell Signaling Technology (Beverly, MA, United States).

### Methods

#### Cell Viability Analysis

3-(4,5-Dimethylthiazol-2-yl)-2,5-diphenyltetrazolium bromide (MTT) assays were used to evaluate the cell growth inhibitory effect of 2-pyridyl cyclohexanone (Hu et al., 2014; Kumar et al., 2018). The concentration of 2-pyridyl cyclohexanone that inhibits cell growth by 50% (IC_50_) after 48 h of treatment was also studied. Cells were seeded into a 96-well plate (4.0 × 10^3^ cells each well) to measure cell proliferation rate. The cells were cultured overnight and incubated with different concentrations of 2-pyridyl cyclohexanone (0, 8, 1.6, or 3.2 μM) for 48 h. Cell viability was assessed by measuring absorbance at 570 nm using a microplate reader (Bio-Rad, Hercules, CA, United States). Experiments were performed in triplicate at least twice.

#### Flow Cytometry and Annexin V-Fluorescein Isothiocyanate (FITC)/Propidium Iodide (PI) Double Staining

Apoptosis was measured with an Annexin V-FITC apoptosis detection kit (KeyGEN, Nanjing, China). Briefly, cells (4 × 10^4^ cells/ml) were incubated with 2-pyridyl cyclohexanone (0, 8, 1.6, or 3.2 μM) for 48 h, centrifuged at 600 × *g* for 5 min, washed twice with cold phosphate-buffered saline (PBS), and resuspended in 100 μl binding buffer. This was followed by staining with 5 μl Annexin V and 5 μl PI in the dark at room temperature 25°C for 15 min. Cells fluorescence was then assayed by flow cytometry (Beckman Coulter Inc., Brea, CA, United States).

#### Evaluation of Mitochondrial Membrane Potential (MMP)

After treatment with different concentrations of 2-pyridyl cyclohexanone for 48 h and washed twice with PBS, cells were incubated with 10 μg/ml JC-1 (Beyotime Institute of Biotechnology, Shanghai, China) for 15 min at 37°C. Then cells were subjected to flow cytometry analysis.

#### Western Blot Analysis

Harvested cells were washed twice in PBS, and lysed in sodium dodecyl sulfate (SDS) lysis buffer containing 1 mM phenylmethylsulfonyl fluoride (PMSF) (PMSF:SDS = 1:50) at 100°C for 30 min. Insoluble cell debris was discarded following centrifugation (12,000 rpm) at 4°C for 15 min (Xu et al., 2016). Cell lysates were separated by SDS–polyacrylamide gel electrophoresis (SDS–PAGE) on 10–12% gels and then transferred onto polyvinylidene membranes (Millipore, Billerica, MA, United States). Immunoblotting was performed for STAT3, p-STAT3, JAK2, p-JAK2, extracellular signal-regulated kinase (ERK), phospho-ERK (p-ERK), p38, phospho-p38 (p-p38), poly (ADP-ribose) polymerase (PARP), Caspase-3, Bcl-xL, Bcl-2, Bax, and Bid, using GAPDH as an internal control.

#### 4’,6-Diamidino-2-Phenylindole (DAPI) Staining Assay

Apoptosis was evaluated by DAPI staining. Cells (4 × 10^4^ cells/ml) were cultured in a confocal dish and treated with 2-pyridyl cyclohexanone for 48 h. Morphological changes were initially evaluated using an inverted light microscope (TH4-200; Olympus, Tokyo, Japan) to detect changes in the nuclei. Treated and untreated cells were fixed in 4% paraformaldehyde at 37°C for 15 min, permeabilized with 0.1% Triton X-100/PBS for 5 min, and incubated with 5 μg/ml DAPI for 15 min (Cui et al., 2016). The cells were washed twice with PBS and photographed using an inverted fluorescence microscopy (TH4-200, Olympus, Tokyo, Japan).

#### Chromatin Immunoprecipitation (ChIP) Assay

Eca109 cells were treated with or without 3.2 μM 2-pyridyl cyclohexanone for 48 h and incubated with 1% formaldehyde at 37°C for 10 min. The harvested cells were resuspended in lysis buffer (10 mmol/l ethylenediaminetetraacetic acid, 1% SDS, and 50 mmol/l Tris–HCl; pH 8.1; Thermo Fisher Scientific, Waltham, MA, United States), incubated at 4°C for 10 min, and then sonicated to produce 100–400 bp DNA fragments. One-third of the lysate was used as the DNA input control. Immunoprecipitated complexes were collected using protein A/G agarose beads, washed, and incubated at room temperature for 20 min. After reversing the cross-linking of the protein–DNA complexes at 65°C for 5 h, DNA was extracted with phenol/chloroform and analyzed by polymerase chain reaction (PCR) using primers specific to the Bcl-2 promoter (Li et al., 2013). ChIP products were also analyzed by PCR. Four pairs of primers were used to amplify 147, 81, 99, and 82 bp fragments associated with regulatory regions of the human Bcl-2 promoter using the following primers: Bcl-2-F1, 5′-TCGTGTAGCACTAAACCAGTG-3′; Bcl-2-R1, 5′-CGTGTCCACCTGAACACCTA-3′; Bcl-2-F2, 5′-GAAGCTACTTGAAAGTAAACACCAC-3′; Bcl-2-R2, 5′-GCTGTGAAGACAGGTGACTCT-3′; Bcl-2-F3, 5′-AGGAGGGCTCTTTCTTTCTTC-3′; Bcl-2-R3, 5′-TGCCTGTCCTCTTACTTCATTC-3′; Bcl-2-F4, 5′-GCGTGTAAATTGCCGAGAAGG-3′; and Bcl-2-R4, 5′-GCGGCGGCAGATGAATTAC-3′. The PCR products were analyzed on a 2% agarose gel and quantified by densitometry using a fluorimeter (Fluor, Irving, TX, United States) and Quantity One software (Bio-Rad).

#### Plasmids Constructs, Transient Transfection, and Dual Luciferase Assay

Human Bcl-2 promoter was cloned into pGL3 plasmid (Promega, Madison, WI, United States) according to the instructions. Bcl-2 promoter was amplified from genomic DNA isolated from Eca109 cells. Mutants were generated by a site-directed mutagenesis kit (Stratagene, Santa Clara, CA, United States). Twenty micrograms of reporter plasmid and 1 μg Renilla luciferase control were co-transfected into Eca109 cells. 48 h post transfection cells were analyzed by using a Dual-Luciferase^®^ Reporter Assay System (Promega, Madison, WI, United States) according to the instructions. Each transfection was performed in triplicate wells and replicated with similar results in at least three independent experiments.

Eca109 cells were cultured in 24-well plates, grown to 80% confluence, and transfected with 0.2 g of luciferase reporter plasmids and 0.2–1.0 g of the indicated plasmids using Lipofectamine^®^ 2000. Next, 0.2 g of 1.6–2.0 μg/ml of the pGL3 vector was co-transfected as the internal control. The amount of DNA in each transfection was kept constant by the addition of an empty expression vector. At 48 h after transfection, luciferase activity was measured using the Dual-Luciferase^®^ Reporter Assay System (GloMaxTM 20/20, Promega, Madison, WI, United States) with a Lumat LB9507 luminometer (Berthold Technologies, Bad Wildbad, Germany) and a TD-20/20 luminometer (Turner Design, Sunnyvale, CA, United States) (Ding et al., 2013). Each transfection was performed in triplicate wells and replicated with similar results in at least three independent experiments.

#### Inhibition of S3I-201 on the Expression of STAT3 in Eca109 Cell

Overexpression experiments were conducted in which reporter plasmids were co-transfected with pcDNA3.1(+) expression vector or a corresponding empty vector. In further experiments, Eca109 cells (40,000/ml) with STAT3 overexpression were incubated with S3I-201 (100 μM), a STAT3 inhibitor, for 48 h to evaluate the inhibitory effect of S3I-201. For western blotting experiments, lysates were obtained from cells cultured for 48 h in 100-mm well plates with GAPDH as an internal control.

### Statistical Analysis

All quantitative data have been presented as mean ± standard deviation (SD) of the results from three independent experiments performed in triplicate. Student’s *t*-test was performed to assess whether there were statistically significant differences in the results. *P*-values < 0.05 were considered statistically significant.

## Results

### Effects of 2-Pyridyl Cyclohexanone on the Growth of Eca109 and EC9706 Cells

**FIGURE 1:**
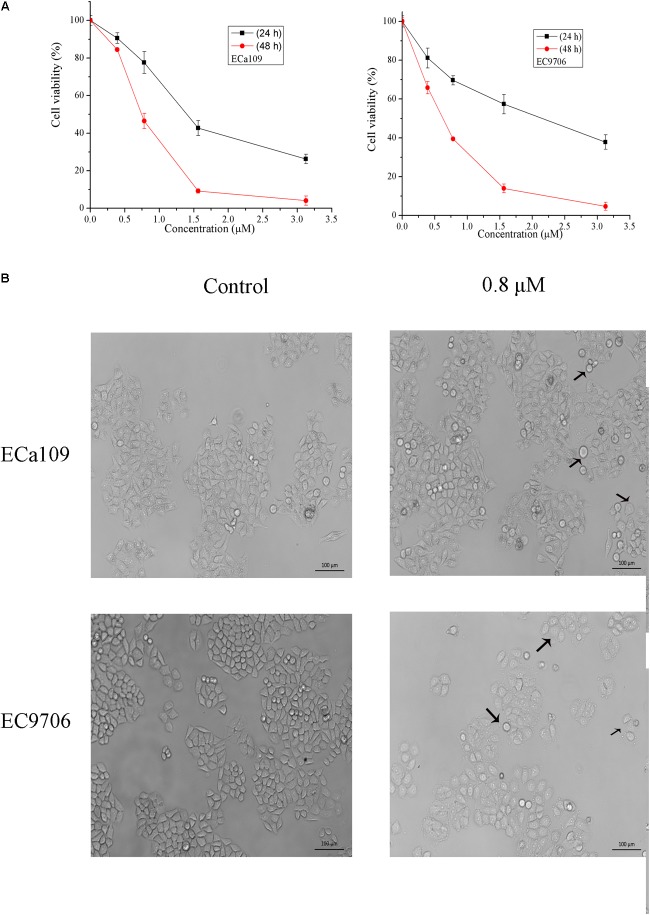
2-Pyridyl cyclohexanone inhibits esophageal cancer cell proliferation. **(A)** Effects of 2-pyridyl cyclohexanone on cell growth. **(B)** Micrographs of Eca109 and EC9706 cells after treatment with 2-pyridyl cyclohexanone. Eca109 and EC9706 cells were treated with the indicated concentrations of 2-pyridyl cyclohexanone for 48 h. Cell viability was quantified by MTT assay. Data are presented as mean ± SD of the results from at least three independent experiments.

To determine the growth inhibitory activity of 2-pyridyl cyclohexanone, human ESCC Eca109 and EC9706 cells were treated with 0, 0.8, 1.6, or 3.2 μM 2-pyridyl cyclohexanone for 24 or 48 h and then viable cells were measured by MTT assay. The results showed that 2-pyridyl cyclohexanone significantly suppressed the growth of the cells in time- and dose-dependent manners. This anti-proliferation effect was observed within a 24-h period; however, it continued to notably increase over the next 48 h (**Figure 1A**). The IC_50_ values of 2-pyridyl cyclohexanone were 1.40 and 0.77 μM against the Eca109 cells, and 2.10 and 0.65 μM against the EC9706 cells.

### 2-Pyridyl Cyclohexanone Induces Apoptosis of Eca109 and EC9706 Cells

**FIGURE 2:**
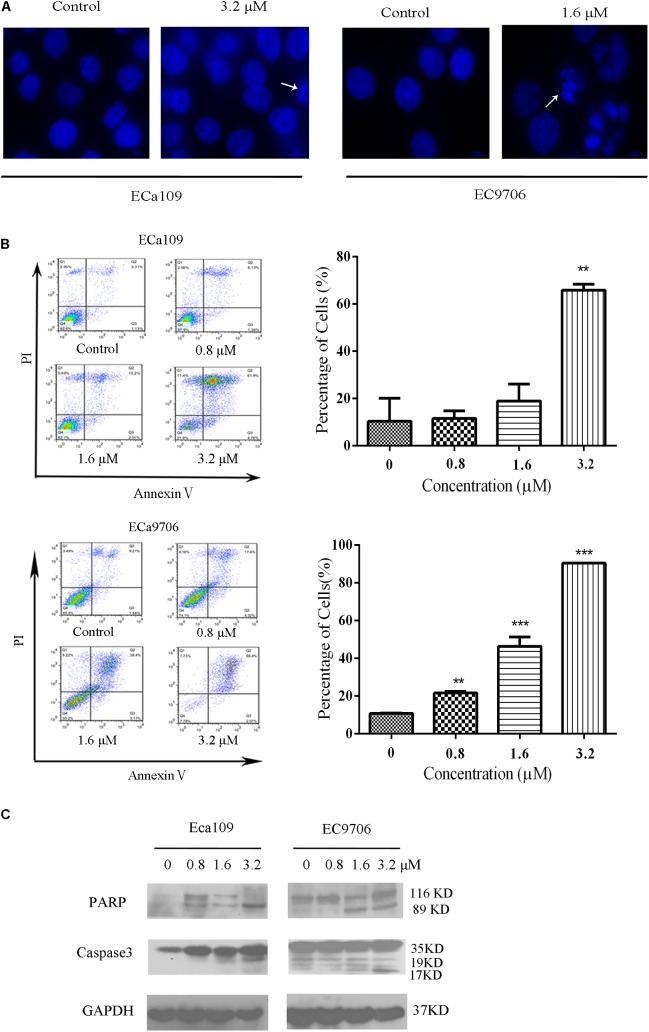
2-Pyridyl cyclohexanone induces apoptosis of Eca109 and EC9706 cells. **(A)** Morphological changes of apoptotic Eca109 and EC9706 cells after treatment with 2-pyridyl cyclohexanone. Eca109 and EC9706 cells were treated with 3.2 and 1.6 μM 2-pyridyl cyclohexanone, respectively, for 48 h. Morphological changes were observed under a fluorescent microscope after DAPI staining. **(B)** Induction of apoptosis by 2-pyridyl cyclohexanone in Eca109 and EC9706 cells. Cells were treated with 0, 0.8, 1.6, or 3.2 μM 2-pyridyl cyclohexanone for 48 h. Apoptosis was assessed by flow cytometry after Annexin V-FITC/PI staining. The percentages of apoptotic cells are indicated as mean ± SD of the results from three independent experiments. Data were analyzed using GraphPad Prism 6.02 software (GraphPad Software Inc., La Jolla, CA, United States). ^∗^ indicates *p* < 0.05, whereas ^∗∗^ indicates *p* < 0.01. **(C)** Western blot analysis of the apoptosis-associated proteins PARP and caspase-3. GAPDH was used as the protein loading control. Data are presented for three independent experiments.

Cell death can generally be divided into three ways, including apoptosis, cell necrosis, and autophagy (Edinger and Thompson, 2004; Hetz, 2008). When cells undergo apoptosis, some significant changes in morphology occur, including chromatin condensation, breakage, and appearance of apoptotic bodies (Ihara et al., 1998). **Figure 1B** showed that the morphology of control was fusiform and in alignment, whereas the morphology of cells treated with 2-pyridyl cyclohexanone became round and some dead cells appeared in the culture fluid. Based on the observed antiproliferative effect of 2-pyridyl cyclohexanone, we investigated whether 2-pyridyl cyclohexanone affects cell progression. We further examined morphological changes in human ESCC cells treated with 2-pyridyl cyclohexanone by DAPI staining. As illustrated in **Figure 2A**, the control cells showed an intact nuclear structure, and the chromatin had a uniform brightness, whereas cells treated with 2-pyridyl cyclohexanone showed bright chromatin, major ultra-microstructural changes such as chromatin aggregation, apoptotic body appeared, and swelling of cytoplasmic compartments. Furthermore, we found EC9706 cells treated with 1.6 μM 2-pyridyl cyclohexanone showed obvious nuclear breakage. It was suggested that 2-pyridylcyclohexanone could induce apoptosis of Eca109 and EC9706 cells, and EC9706 was more sensitive to drugs.

In the apoptosis study, cells were stained with Annexin V-FITC/PI and analyzed by flow cytometry (**Figure 2B**). We found that 2-pyridyl cyclohexanone significantly induced apoptosis of Eca109 and EC9706 cells. At 48 h after treatment, the total apoptotic rates of Eca109 cells were found to be 11.5, 18.9, and 65.8%, respectively, at 2-pyridyl cyclohexanone concentrations of 0.8, 1.6, and 3.2 μM. Furthermore, at concentrations of 1.6 and 3.2 μM 2-pyridyl cyclohexanone caused cell death as indicated by Annexin V-FITC and Annexin V-FITC/PI staining. In the Eca109 cells, 2-pyridyl cyclohexanone caused an increase in apoptotic cells at concentrations of 1.6 and 3.2 μM; however, it caused a relatively large increase in apoptotic cells (65.8%) at 3.2 μM. Similarly, in the EC9706 cells, the percentage of apoptotic cells was 21.5, 46.3, and 90.4% after the treatment with 2-pyridyl cyclohexanone. These findings indicate that 2-pyridyl cyclohexanone inhibits the growth of Eca109 and EC9706 cells by inducing apoptosis in a dose-dependent manner.

Further confirmation that the cells were undergoing apoptosis was obtained by western blot analyses for caspases 3 and its substrate PARP proteins in the cell lines treated with 0.8, 1.6, or 3.2 μM 2-pyridyl cyclohexanone for 48 h. As seen in **Figure 2C**, 2-pyridyl cyclohexanone could induce the activation of caspase 3. Consistent with the activation of caspase 3, proteolytic cleavage was found in PARP, which was evident by the presence of 116–89 kDa fragments in both cell lines. In the EC9706 cells, an increase in the amount of 89-kDa PARP fragments could readily be seen after the treatment with 2-pyridyl cyclohexanone. These results suggest that 2-pyridyl cyclohexanone is a potent inducer of apoptosis in ESCC cells.

### 2-Pyridyl Cyclohexanone Induces Apoptosis Through the Mitochondria-Mediated Intrinsic Pathway

**FIGURE 3:**
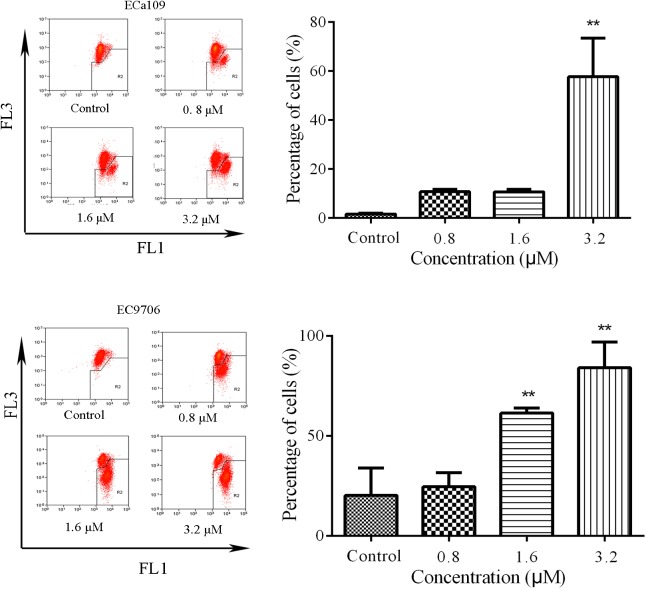
Effects of 2-pyridyl cyclohexanone on MMP. Flow cytometry analysis of MMP by JC-1 staining. Cells were treated with 0, 0.8, 1.6, or 3.2 μM 2-pyridyl cyclohexanone for 48 h, and cells with MMP loss were gated. Data are presented as mean ± SD of the results from three independent experiments. ^∗^ indicates *p* < 0.05, whereas ^∗∗^ indicates *p* < 0.01.

Reduction in MMP is indicative of early apoptosis. JC-1 is widely used in the detection of MMP. When MMP is high, JC-1 gathers in the mitochondrial matrix, which results in the formation of polymer J-aggregates and a red fluorescence. However, when MMP is low, JC-1 does not gather in the mitochondrial matrix. It therefore exists as a monomer and produces a green fluorescence (Reers et al., 1995). These color changes are used to easily detect changes in MMP (Δψm). Therefore, we measured MMP in the Eca109 and EC9706 cells after treatment with 2-pyridyl cyclohexanone using the membrane-permeable JC-1 dye. As shown in **Figure 3**, a marked decrease in red fluorescence was seen in both cell lines after the treatment with 3.2 μM of 2-pyridyl cyclohexanone. These results demonstrate that 2-pyridyl cyclohexanone disrupts MMP in both cell lines.

### 2-Pyridyl Cyclohexanone Regulates the Expression of Bcl-2 Family Proteins

**FIGURE 4:**
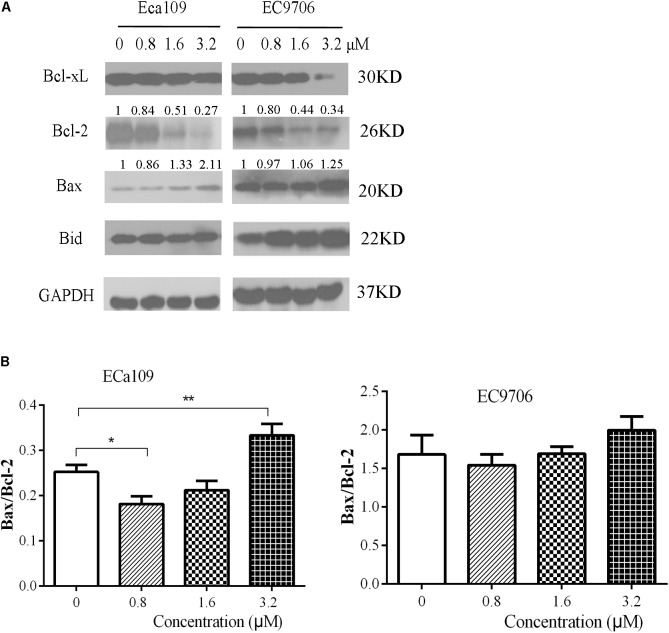
Effects of 2-pyridyl cyclohexanone on Bcl-2 family proteins. **(A)** Bcl-xL, Bcl-2, Bid, and Bax protein levels were assessed by western blotting in Eca109 and EC9706 cells after treatment for 48 h with 2-pyridyl cyclohexanone. GAPDH was used as an internal control. **(B)** Bax/Bcl-2 ratio in Eca109 and EC9706 cells. Data are presented for three independent experiments.^∗^ indicates *p* < 0.05, whereas ^∗∗^ indicates *p* < 0.01.

Further confirmation that the cells were undergoing apoptosis was obtained by western blot analyses for anti-apoptotic Bcl-2/Bcl-xL and pro-apoptotic Bax/BID proteins. As shown in **Figure 4A**, 2-pyridyl cyclohexanone downregulated Bcl-2 and Bcl-xL protein expression but upregulated Bax and Bid protein expression in ECa109 cells and EC9706 cells. In addition, it significantly increased the Bax/Bcl-2 ratio in ECa109 cells treated with 3.2 μM 2-pyridyl cyclohexanone for 48 h (**Figure 4B**). Taken together, the results show that 2-pyridyl cyclohexanone disrupts MMP, disturbs the balance of the Bcl-2 family proteins, and triggers apoptosis via the mitochondria-mediated intrinsic pathway.

### Effect of 2-Pyridyl Cyclohexanone on Mitogen-Activated Protein Kinase (MAPK) Signaling in Eca109 and EC9706 Cells

**FIGURE 5:**
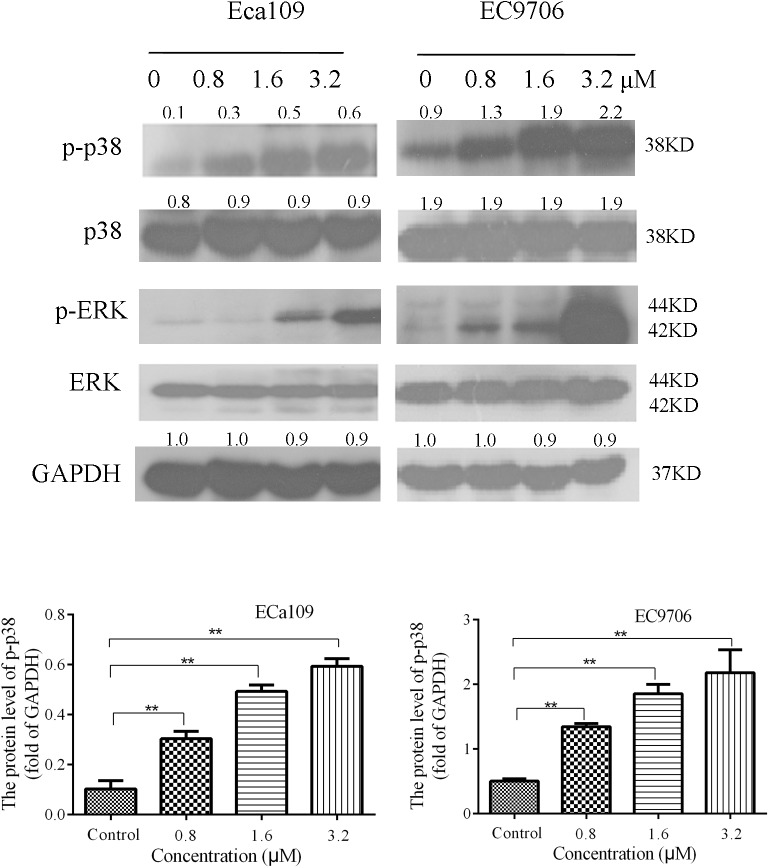
Effects of 2-pyridyl cyclohexanone on p38/ERK MAPK expression in Eca109 and EC9706 cells. Cells were treated with 0, 0.8, 1.6, or 3.2 μM 2-pyridyl cyclohexanone for 48 h and subjected to western blot analysis. GAPDH was used as the internal control. Data are presented for three independent experiments.

The MAPK signaling pathway (ERK, p38) is involved in the regulation of cell growth and differentiation, environmental adaptation to stress, and cancer progression (Long et al., 2014). Therefore, we tested the effect of 2-pyridyl cyclohexanone on proteins involved in MAPK signaling pathways. The results showed that the levels of phosphorylated p38 and phosphorylated ERK increased significantly in a dose-dependent manner (**Figure 5**). This suggests that 2-pyridine cyclohexanone activates MAPK signals in Eca109 and EC9706 cells. In addition, the MAPK signaling pathway may be involved in apoptosis induced by 2-pyridine cyclohexanone.

### 2-Pyridyl Cyclohexanone Decreases the Phosphorylation of STAT3 and JAK2 in Eca109 and EC9706 Cells

**FIGURE 6:**
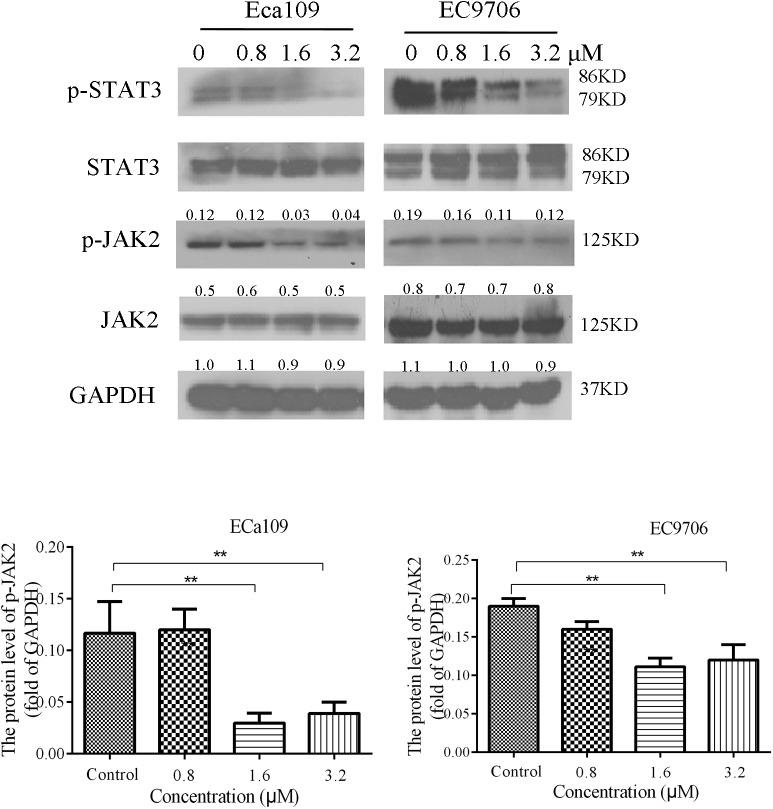
2-Pyridyl cyclohexanone inhibits the STAT3 signaling pathway. Eca109 and EC9706 cells were treated with 0, 0.8, 1.6, or 3.2 μM 2-pyridyl cyclohexanone for 48 h. The expression levels of STAT3, p-STAT3, JAK2, and p-JAK2 were determined by western blot analysis. GAPDH was used as the internal control. Data are presented for three independent experiments.

STAT3 is aberrantly activated in various types of malignancies. In addition, it plays crucial roles in tumor cell proliferation and survival, and tumor angiogenesis and invasion (Horiguchi et al., 2002; Masuda et al., 2009). Eca109 and EC9706 cells were treated with 2-pyridyl cyclohexanone (0, 0.8, 1.6, or 3.2 μM) for 48 h to analyze the effects of the latter on phosphorylation of STAT3 and JAK2. The results showed a dose-dependent decrease in the phosphorylation of STAT3 and JAK2, as was demonstrated by western blotting (**Figure 6**). It was also found that EC9706 cells were more sensitive than Eca109 cells were to the same concentration of 2-pyridyl cyclohexanone.

### Effect of Inhibited Expression of STAT3 on Bcl-2 Expression in Eca109 Cells

**FIGURE 7:**
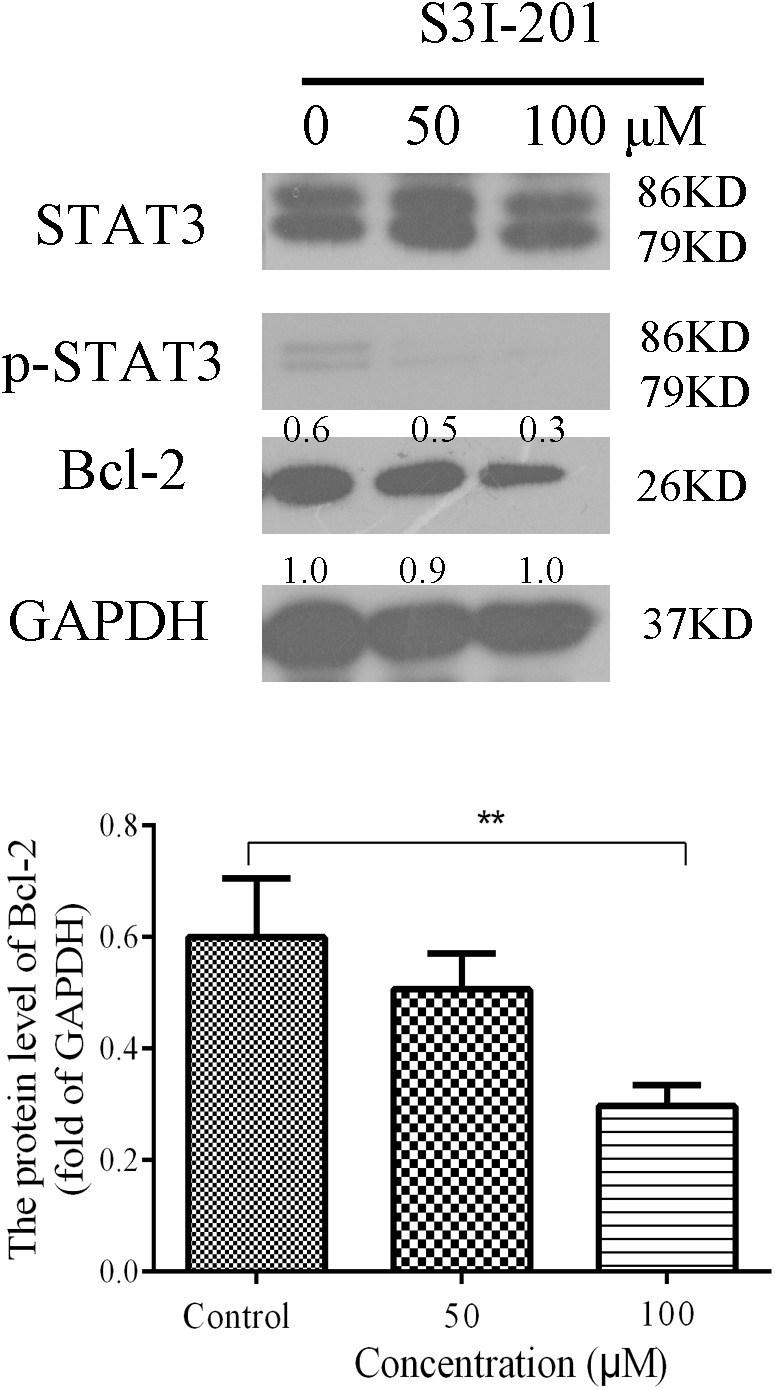
Effect of S3I-201 on Eca109 cells. Eca109 cells were treated with 50 or 100 μM S3I-201 for 48 h, after which the expression levels of p-STAT3, STAT3, and Bcl-2 were measured by western blot analysis. GAPDH was used as the internal control. Data are presented for three independent experiments.

We further examined whether inhibiting the expression of STAT3 in Eca109 cells could influence Bcl-2 expression. Eca109 cells were incubated with 50 or 100 μM S3I-201 (STAT3 inhibitor) for 48 h, after which Bcl-2 expression was evaluated by western blotting. As illustrated in **Figure 7**, the expression levels of p-STAT3 and Bcl-2 reduced in the Eca109 cells. The results indicated that STAT3 could be an effective target in ESCC treatment.

### STAT3 Activates Bcl-2 Transcription in Eca109 Cells

**FIGURE 8:**
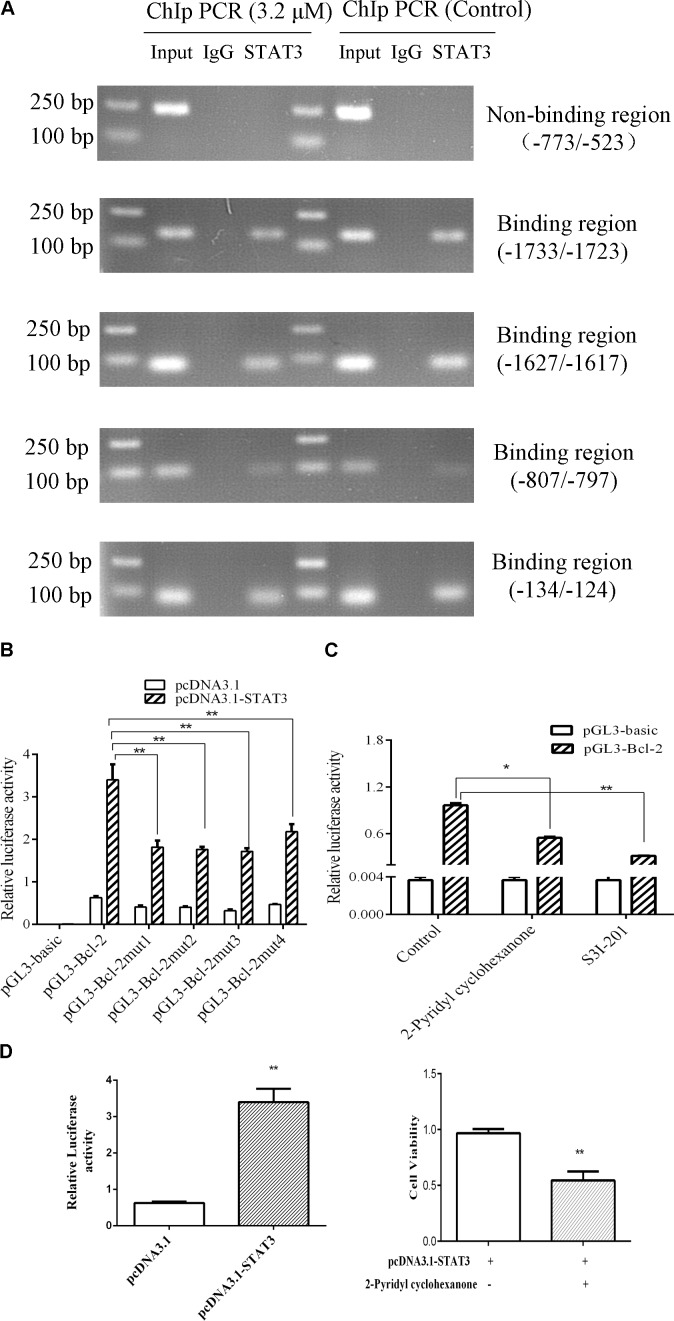
STAT3 activates Bcl-2 transcription in Eca109 cells. **(A)** The binding of STAT3 to Bcl-2 promoter was analyzed in a ChIP assay. Input DNA was used as a positive control. **(B)** The transcription activities of Bcl-2 were analyzed following overexpression of STAT3 vector in Eca109 cells (^∗∗^ indicates *p* < 0.01). The cells were co-transfected with pcDNA3.1-STAT3 vector, and Renilla luciferase plasmid was used as a control. **(C)** The transcription activities of Bcl-2 were analyzed following treatment of the cells with 3.2 μM 2-pyridyl cyclohexanone and 100 μM S3I-201. S3I-201 was used as the positive control. All data are presented as mean ± SD of the results from three experiments. ^∗^ indicates *p* < 0.05, whereas ^∗∗^ indicates *p* < 0.01. **(D)** ECa109cells were co-transfected with pcDNA3.1-STAT3 vector. The effect of proliferation on ECa109 cells treated with 3.2 μM 2-pyridyl cyclohexanone for 48 h were to analyzed (^∗∗^ indicates *p* < 0.01).

To verify whether STAT3 interacts with Bcl-2 promoter, we conducted ChIP assays in Eca109 cell with anti-STAT3 antibodies. As shown in **Figure 8A**, STAT3 was proved to bind to the promoter of Bcl2 in the Eca109 cells via ChiP experiment. **Figure 8A** showed point mutations created in Bcl-2. As shown in **Figure 8B**, we observed the relative luciferase activity was 1.82, 1.76, 1.71, and 2.18 in promoter activity in the elements carrying each mutant region (Bcl-2), as compared with the wild-type promoter (3.40) in the Eca109 cells. It was 51.87, 48.19, 49.55, and 35.91% decreases. In general, these results revealed that STAT3 has a predominant role in the transcriptional regulation of Bcl-2 promoter activity.

To further investigate the roles of STAT3 in the regulation of Bcl-2 promoter activity, ectopic overexpression of pcDNA3.1-STAT3 co-transfected into Eca109 cells was investigated. The results showed that luciferase activity was distinctly lower in cells transfected with the empty pcDNA3.1 vector control (**Figure 8B**). S3I-201 blocks STAT3 function in cancer cells by binding to the STAT3 SH2 domain to disrupt STAT3 protein complexation events (Ball et al., 2016). Promoter activity was significantly attenuated in the Eca109 cells when each site was mutated separately (**Figure 8B**). **Figure 6** showed Bcl-2 was downregulated after treatment with 2-pyridyl cyclohexanone for 48 h. Moreover, the dual luciferase assay demonstrated that 2-pyridyl cyclohexanone repressed STAT3 at the transcription level (**Figure 8C**), S3I-201 as positive control. **Figure 8D** showed that 3.2 μM 2-pyridyl cyclohexanone could significantly inhibit cell proliferation under the condition of STAT3 overexpression. Taken together, these results indicate that STAT3 directly binds to the predicted sites in the Bcl-2 promoter region and is crucial for the transcriptional activation of Bcl-2 expression.

## Discussion

The transcription factor STAT3 promotes cell proliferation and angiogenesis; however, it inhibits apoptosis of malignant cells. Importantly, constitutive STAT3 activation has been documented in several tumor types and is correlated with tumorigenesis. Moreover, it is considered to be an oncogene (Bromberg et al., 1999) in human renal cell carcinoma and has a negative impact on prognosis (Horiguchi et al., 2002; Masuda et al., 2009). The advantages of targeting STAT3 in cancer therapy have been fully investigated. Accumulating evidences have shown that inhibition of constitutively active STAT3 leads to impaired cell survival and proliferation (Yu and Jove, 2004; Frank, 2007; Yue and Turkson, 2009). The capability of curcumin to inhibit the JAK2/STAT3 pathway makes it a desirable lead compound (Lin et al., 2010b; Abroun et al., 2015). Thus, the abovementioned molecular pathway is attractive in designing small molecule inhibitors (Yu et al., 2007; Yue and Turkson, 2009).

Curcumin is a recognized diarylheptanoid constituent of turmeric that has shown antitumous effects under pre-clinical and clinical conditions (Kasi et al., 2016). In addition, it is distinguished that the antitumous effects of curcumin are chiefly due to activation of apoptotic pathways in malignancy cells and inhibition of inflammation, angiogenesis, and metastasis in tumor microenvironments. Particularly, numerous studies have demonstrated that curcumin targets many therapeutically important cancer signaling pathways such as STAT3 (Blasius et al., 2006; Chakravarti et al., 2006), HER2 (Aggarwal et al., 2003), Notch (Subramaniam et al., 2012), p53, Ras, Wnt-β catenin (Leow et al., 2010), NF-κB (Epstein et al., 2010), phosphatidylinositol-4,5-bisphosphate 3-kinase, AKT (Chiablaem et al., 2014), and mechanistic target of rapamycin (Seo et al., 2014) signaling pathways, among others. It was demonstrated that curcumin could inhibit Ec109 cell growth via an AMPK-mediated metabolic switch, and it could cause a significant down-regulation of glycolytic enzymes expressions in a dose-dependent manner (Zhang et al., 2015). It also could inhibit colony formation and induce cell death through modulating notch signaling in esophageal cancer cells (Subramaniam et al., 2012). Moreover, curcumin could suppress vasculogenic mimicry capacity of hepatocellular carcinoma cells through STAT3 and PI3K/AKT inhibition (Chiablaem et al., 2014). Nevertheless, after oral administration blood levels of curcumin are low, and this may limit its clinical usage (Sharma et al., 2007). Many studies attempt to improve its chemical properties by variouse structure modifications (Lin et al., 2011; Zhou et al., 2014a,b; Dong et al., 2016).

In the present study, we explored the effect of 2-pyridyl cyclohexanone on STAT3 DNA (*Bcl-2*) binding activity and expression, and investigated signal transduction pathways involved in apoptosis and the growth of ESCC cells. We found that treatment of human ESCC cell lines with 2-pyridyl cyclohexanone promoted loss of both p-STAT3 and p-JAK2. Based on these findings, the relationship between STAT3 and apoptosis in these cells was investigated. The results from the analysis of cleaved PARP levels showed that 2-pyridyl cyclohexanone induced apoptosis in the cells following their exposure to the compound. PARP is an early target of active caspases. In addition, the product of its cleavage serves as a marker of apoptosis. Western blot analysis of cleavage-specific PARP antibody revealed that 2-pyridyl cyclohexanone induced apoptosis of Eca109 cells at concentrations as low as 1.6 and 3.2 μM (**Figure 2C**). Cleaved PARP was also detected in the EC9706 cell line at lower concentrations of 2-pyridyl cyclohexanone. To determine if downregulation of p-STAT3 decreases apoptosis in esophageal carcinoma cells, we further investigated the levels of proteins involved in the MAPK signaling pathway. This indicates that the MAPK signaling pathway may be involved in the apoptosis induced by 2-pyridyl cyclohexanone.

Mitochondria is the central player in cell apoptosis (Reed et al., 1998). Bcl-2 family members play important roles in this pathway (Kuwana et al., 2002; Schwarz et al., 2007). We found that 2-pyridyl cyclohexanone decreased Δψm in the Eca109 and EC9706 cells (**Figure 3**) and suppressed Bcl-2 and Bcl-xL expression, whereas promoted Bid/Bax expression in a dose-dependent manner (**Figure 4**). These findings indicate that loss of Δψm plays an important role in 2-pyridyl-cyclohexanone-induced apoptosis of esophageal cancer cells.

In the present study, we demonstrated that inhibition of STAT3 phosphorylation by 2-pyridyl cyclohexanone results in decreased Bcl-2 expression, increased Bax expression, and induction of apoptosis. These results suggest that STAT3 inhibition by 2-pyridyl cyclohexanone increased the expression of Bcl-2 family proteins. Studies have indicated that *Bcl-2* is a target gene of STAT3 (Abroun et al., 2015). Howerver, it has remained unclear that what exact relationship between Bcl-2 and STAT3. In the ChIP assay, we predicted the gene loci of the *Bcl-2* promoter region. The results showed that there were four STAT3-binding sites in the Bcl-2 promoter region. The ChIP experiments and luciferase assays indicate that promotor regions of Bcl-2 (−1733/−1723), Bcl-2 (−1627/−1617), Bcl-2 (−807/−797), and Bcl-2 (−134/−124) were binding sites for STAT3.

The effect of STAT3 on Bcl-2 was obvious in the Eca109 cells that were treated with 3.2 μM 2-pyridyl cyclohexanone. Luciferase activity changed from 0.97 to 0.55 after the treatment with 2-pyridyl cyclohexanone for 48 h (**Figure 8C**). Therefore, we speculate that 2-pyridyl cyclohexanone induced apoptosis by inhibiting STAT3 phosphorylation in the Eca109 cells. The results of the western blot analysis corroborated this assumption. As described in **Figure 7**, the expression levels of Bcl-2 reduced when we reduced the STAT3 expression levels with S3I-201 (STAT3 inhibitor). Moreover, **Figure 8D** showed that cell proliferation under the condition of STAT3 overexpression could significantly inhibited by 2-pyridyl cyclohexanone.

Our findings indicate that targeting STAT3 may be valuable in the control of cell proliferation in ESCC cells. Certainly, our view also needs further confirmation *in vivo*. The *in vivo* effects of 2-pyridyl cyclohexanone will be the focus of our next study. The specific etiology and pathogenesis of esophageal carcinoma are not clear yet. Significant familial aggregation has been reported to be involved in the development of esophageal carcinoma (Liao et al., 2011). Changes in several related genes have been noted to occur in some families with a high incidence of cancer; however, research on genetic susceptibility to cancer is increasing.

## Conclusion

2-Pyridyl cyclohexanone inhibits STAT3 activation in mediating the expression of Bcl-2 genes to influence the proliferation and survival of ESCC cells. Our results demonstrate that the curcumin analog 2-pyridyl cyclohexanone decreases basal STAT3 phosphorylation and promotes apoptosis of ESCC cells. These data support the view that STAT3 is a related target for therapeutic intervention in ESCC management. This study indicated that STAT3 is a potential therapeutic target in ESCC.

## Author Contributions

YinW carried out most of the studies, designed the study, wrote the manuscript, and offer some suggestions. PZ, DX, SQ, and YL analyzed the results and revised the manuscript and results. WF, BR, LZ, and YZ provided technical assistance with several protocols. XyW, QL, XW, YP, and HY read and revised the entire manuscript. SW and JQ performed data analysis and interpretation. XyW, QL, ZL, and ZD conceived the study, planned the project, and coordination of the study. All authors read and approved the final manuscript.

## Conflict of Interest Statement

The authors declare that the research was conducted in the absence of any commercial or financial relationships that could be construed as a potential conflict of interest.
